# Impact of Co^2+^ Substitution on Microstructure and Magnetic Properties of Co_x_Zn_1-x_Fe_2_O_4_ Nanoparticles

**DOI:** 10.3390/nano9111602

**Published:** 2019-11-11

**Authors:** W. S. Mohamed, Meshal Alzaid, Mohammed S. M. Abdelbaky, Zakariae Amghouz, Santiago García-Granda, Ahmed M. Abu-Dief

**Affiliations:** 1 Physics Department, College of Science, Jouf University, Al-Jouf, Sakaka P.O. Box 2014, Saudi Arabia; mmalzaid@ju.edu.sa; 2 Physics Department, Faculty of Science, Sohag University, 82524 Sohag, Egypt; 3 Departments of Physical and Analytical Chemistry, and Organic and Inorganic Chemistry, University of Oviedo-CINN, 33006 Oviedo, Spain; 4 Department of Materials Science and Metallurgical Engineering, University of Oviedo, Campus Universitario, 33203 Gijón, Spain; 5 Chemistry Department, Faculty of Science, Sohag University, 82524 Sohag, Egypt; 6 Chemistry Department, Faculty of Science, Taibah University, Madinah P.O. Box 344, Saudi Arabia

**Keywords:** Co_x_Zn_1-x_Fe_2_O_4_ nanoparticles, hydrothermal method, magnetic parameters, electron microscopies, ferromagnetic ordering

## Abstract

In the present work, we synthesized Co_x_Zn_1-x_Fe_2_O_4_ spinel ferrite nanoparticles (x= 0, 0.1, 0.2, 0.3 and 0.4) via the precipitation and hydrothermal-joint method. Structural parameters were cross-verified using X-ray powder diffraction (XRPD) and electron microscopy-based techniques. The magnetic parameters were determined by means of vibrating sample magnetometry. The as-synthesized Co_x_Zn_1-x_Fe_2_O_4_ nanoparticles exhibit high phase purity with a single-phase cubic spinel-type structure of Zn-ferrite. The microstructural parameters of the samples were estimated by XRD line profile analysis using the Williamson–Hall approach. The calculated grain sizes from XRPD analysis for the synthesized samples ranged from 8.3 to 11.4 nm. The electron microscopy analysis revealed that the constituents of all powder samples are spherical nanoparticles with proportions highly dependent on the Co doping ratio. The Co_x_Zn_1-x_Fe_2_O_4_ spinel ferrite system exhibits paramagnetic, superparamagnetic and weak ferromagnetic behavior at room temperature depending on the Co^2+^ doping ratio, while ferromagnetic ordering with a clear hysteresis loop is observed at low temperatures (5K). We concluded that replacing Zn^2+^ ions with Co^2+^ ions changes both the structural and magnetic properties of ZnFe_2_O_4_ nanoparticles.

## 1. Introduction

The microstructural characterization of spinel-ferrites has been long discussed in the literature [1,2,3,4]. Such interests are justified by the potential applications of spinel-ferrites that involve spintronic and magnetic resonance imaging (MRI), gas sensors, magnetic recording, medical diagnostics, antibacterial agents and self-controlled magnetic hyperthermia [5,6,7,8]. Thanks to the spinel-ferrites’ exceptional electric and magnetic properties, they are promising significant future advancements in Lithium-Ion batteries, microwave electronics and catalysis [9,10,11]. The coercivity, remanent magnetization, saturation magnetization and anisotropy constant of the spinel-ferrites are found at the basis of many other applications [12].

The spinel ferrite structure features a cubic cell with eight formula units. The formula unit is denoted by (D)**_A_**(T)**_B_**O_4_, where D is the divalent transition metal cation, that is, D = Co, Ni, Mn, Cu, Zn, Mg. T is the iron trivalent cation, that is, Fe^3+^. Inside the cubic cell, the D^2+^ and Fe^3+^ metallic cations are positioned at two distinct interstitial sites (tetrahedral-A or octahedral-B site) [13,14,15,16].

The physicochemical properties of zinc and cobalt ferrites have been at the forefront of advanced research because of their potential use in medical and industrial applications [17,18,19,20]. The structure of ZnFe_2_O_4_ bulk compound is normal spinel ferrite. The diamagnetic Zn^2+^ ions occupancy of the tetrahedral A-sites renders all Fe^3+^ ions on the octahedral B-sites [21]. Moreover, ZnFe_2_O_4_ is a soft magnetic oxide due to the nonmagnetic properties of Zn^2+^ ions (0 μ_B_). On the hand, the Co-based spinel ferrite (CoFe_2_O_4_) is a hard-magnetic oxide due to the large magnetic moments of Co^2+^ ions (3 μ_B_). Cobalt ferrites exhibit a high coercivity (H_C_), moderate magnetization (M_s_) and fine chemical stability. In addition, CoFe_2_O_4_ has an inverse spinel ferrite structure in which ferric ions are equally-distributed on the octahedral and tetrahedral sites. On the other hand, the divalent cobalt ions occupy the octahedral B-sites.

Scientists found that mixing Co–Zn ferrites compounds would result in a mixed spinel ferrite type structure [22]. Furthermore, the magnetic behavior of ZnFe_2_O_4_ is highly dependent on the distribution of the cations of the tetrahedral A sites and octahedral B sites in the spinel structure. Thus, the introduction of magnetic Co^2+^ ions changes the cation distribution in the host ferrite lattice and changes the properties of the parent ferrite material by modifying the super-exchange coupling of the tetrahedral and octahedral lattice sites [23,24,25,26]. Fabrication processes may be controlled to optimize the magnetic properties of spinel ferrite materials for specific uses. This reflects the interest in size-controlled synthesis of ferrite nanomaterials with limited size distributions and a variety of morphologies [27]. For example, Huma et al. [28] studied the synthesis and characterizations of Co^2+^ ions substituted zinc ferrites Co_1-x_Zn_x_Fe_2_O_4_ with different Co^2+^ doping ratios (x = 0.0–1.0) synthesized via the micro-emulsion technique. This research group observed that the crystalline size decreased from 41 nm to 19 nm as the Co^2+^ content increased from x = 0.0 to x = 1.0. They reported a ferromagnetic order at room temperature for all samples with an improvement in coercivity and saturation magnetization by increasing the concentration of Co^2+^ in the host structure. These results contradict other findings obtained by Gomea-Polo et al. [29], who investigated the same nanoferrite system prepared by the co-precipitation method. Their structure and magnetic study indicated that the particle size increases from 9.4 nm to 14.7 nm with the increase of the Co^2+^ content from 0.1 to 1.0, with superparamagnetic behavior at room temperature.

F. Gozuak et al. [30] reported the preparation of Co_1-x_Zn_x_Fe_2_O_4_ nanoferrites (x = 0.0–1.0) via PEG-assisted hydrothermal rout. In this study, the size of particles did not change linearly with the Co^2+^ doping ratio. The particle size decreased from 12.1 nm to 5.3 nm with the increase of the Co^2+^ content from 0.0 to 0.5 and then increased to 12.4 nm with an increase of Co^2+^ content to 0.7. Magnetic measurements showed transformation of magnetic configuration from superparamagnetic to ferromagnetic at room temperature as a result of introducing Co^2+^ ions in the ZnFe_2_O_4_ host lattice.

In this study, we used the precipitation and hydrothermal-joint method to prepare fine well-crystallized Co^2+^ doped zinc ferrite nanopowders. We found that this joint-method is favorable as it guarantees good physical homogeneity, narrow distribution of particle size and high production rates with low reaction temperature. Until now, there have been few studies on the magnetic behavior of Co^2+^ doped zinc ferrite nanoparticles below curie-temperature (T_C_) [31]. Thus, our work fulfills a need to investigate the magnetic properties of Co_1-x_Zn_x_Fe_2_O_4_ with different Co^2+^ doping ratios (0%, 10%, 20%, 30% and 40%) between 5K and 300K. Here, we studied the influence of various Co^+2^ doping ratios on the structure and magnetic properties of ZnFe_2_O_4_ nanomaterial and the corresponding interactions between the Fe^3+^ and Co^2+^ ions during the evolution process.

## 2. Materials and Methods

Co-doped zinc ferrites Co_1-x_Zn_x_Fe_2_O_4_ nanoparticles with different Co^2+^ doping ratios (0%, 10%, 20%, 30% and 40%) (Co_ (0, 1, 2, 3 and 4)) were synthesized by using stoichiometric molar amounts of Co (NO_3_)_2_·6H_2_O, Zn(NO_3_)_2_·6H_2_O and Fe(NO_3_)_3_·9H_2_O obtained from Sigma Aldrich Corporation (Germany). These materials were chromium, zinc and iron precursors, while NaOH pellets obtained from Alfa Aesar Corporation was the precipitating agent, and Polyethylene glycol 400 was used as a surfactant. Co_ (0, 1, 2, 3 and 4) samples were prepared via the precipitation and hydrothermal-joint method. The details of the sample preparation procedures have been presented in our previous work [21]. X-ray powder diffraction (XRPD) statistics of Co-doped zinc ferrites Co_1-x_Zn_x_Fe_2_O_4_ nanoparticles were investigated by a Philips X’pert MRD diffractometer with Cu Kα radiation wavelength of 1.5418 Å in the 2-Theta range from 10° to 80°. The energy dispersive X-ray spectroscopy (EDXS) analysis was carried out with a scanning electron microscope (SEM/EDXS-JEOL- 6610VL) operating at 2000 V. Transmission electron microscopy (TEM) and high-resolution HR-(TEM) investigations were performed with a JEOL-JEM-2100F microscope operating at 200 kV. The magnetic properties of the Co_x_Zn_1-x_Fe_2_O_4_ ferrite systems were measured using quantum design vibrating sample magnetometer (VSM) within the range of ± 50,000 Oe between 5 and 300 K.

## 3. Results and Discussion

### 3.1. XRPD Analysis

#### 3.1.1. Phase Identification

**Figure 1:**
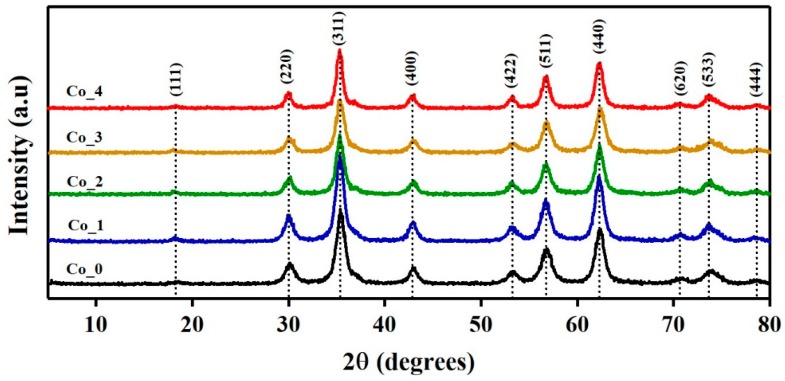
X-ray powder diffraction (XRPD) patterns of Co_x_Zn_1-x_Fe_2_O_4_ ferrite systems.

The crystal structure of the synthesized Co_ (0, 1, 2, 3 and 4) samples with composition Co_x_Zn_1-x_Fe_2_O_4_ (x = 0, 0.1, 0.2, 0.3 and 0.4), respectively, were analyzed using the XRPD technique. The XRPD patterns of the Co_x_Zn_1-x_Fe_2_O_4_ samples with different Co doping ratios, i.e., 0–40% are shown in Figure 1.

The well-defined sharp diffraction peaks of all the samples indicates a high degree of crystallization of Co_x_Zn_1-x_Fe_2_O_4_ crystals. The observed peaks of the XRPD spectra features the expected Bragg peak solely for a single-phase spinel-type cubic structure of Zn-ferrite with *Fd-3m* space group (JCPDS file no. 022-1012). The absence of secondary crystalline phases reveals the high purity of the synthesized samples. This shows that Co atoms were successfully substituted into the lattice of Zn-ferrite. Table S1 summarizes the detected XRPD lines, their (*2θ*) angles, the calculated interplanar distances (*d_hkl_*), Miller indices (*hkl*) and the calculated pure broadening (*β_Correct_)* for all samples. The interplanar distance values were calculated according to Bragg’s law:

(1) $$n\lambda =2d_{hkl}\;sin\;\theta ,$$

where *n* is the order of diffraction, *λ* is the X-ray wavelength and *d_hkl_* is the lattice spacing.

#### 3.1.2. Microstructural Studies by Williamson–Hall (W–H) Method

Analyzing the X-ray peak profiles with the (W–H) method enables estimation of the crystallite size (i.e., coherently diffracting domains) and the lattice strain (i.e., the lattice parameters arising from crystal imperfections) by studying the peak width as a function of detector angle 2-Theta [32,33].

A Lorentzian model was used to fit the individual diffraction peaks for all samples using Origin 8.1 software and Lorentz function in the fitting procedure. According to the Lorentzian distribution function, the corrected broadening of a peak *β_Correct_* is related to the observed broadening *β_Observed_* and the instrumental broadening *β_Instrumental_* by the following relation:

(2) $$\beta_{Correct}=\beta_{Observed}-\beta_{Instrumental}$$

The observed line integral breadth was used to calculate the pure broadening of XRPD lines of our investigated samples. After the Lorentzian fitting of the spectral XRPD peaks in Figure S1, the line integral breadth for a specific peak is calculated from the integral normalized by the maximum intensity of that peak. The XRPD profile of a highly crystalline LaB_6_ (660a) was used as a Standard Reference Material (SRM) to calculate the instrumental broadening correction, in which the impact on broadening due to particle size is minimal. Table S1 reports the calculated values of the corrected broadening *β_Correct_* for the XRD peaks.

According to the W–H approach, the broadening of the diffraction peaks is due to crystallite size (*β_D_*) and micro-strain (*β_ε_*) contributions. Thus, the pure peak broadening (*β_Correct_*) for the samples in under study read [34] as

(3) $$\beta_{Correct}=\beta_{D}-\beta_{\varepsilon }=\frac{k\lambda }{D_{L}\;cos\theta }+4\varepsilon_{L}\;tan\theta ,$$

(4) $$\beta_{Correct}\;cos\theta =\frac{k\lambda }{D_{L}\;}+4\varepsilon_{L}\;sin\theta$$

where *k* is the shape factor (~0.94), *λ* is the incident XRPD wavelength (~0.15418 nm), *D_L_* is the average crystallite size, and *ε_L_* is the average micro-strain. Equation (4) assumes that the sample has an isotropic nature and the micro-strain is uniform in all (*hkl*) crystallographic planes. This equation represents the uniform deformation model (UDM) where the crystal is considered isotropic in nature [32,33].

The average crystallite size (*D_L_*) and the average micro-strain (*ε_L_*) for all Co_ (0, 1, 2, 3 and 4) samples were calculated with Equation (4) and the results are in Table 1.

**Figure 2:**
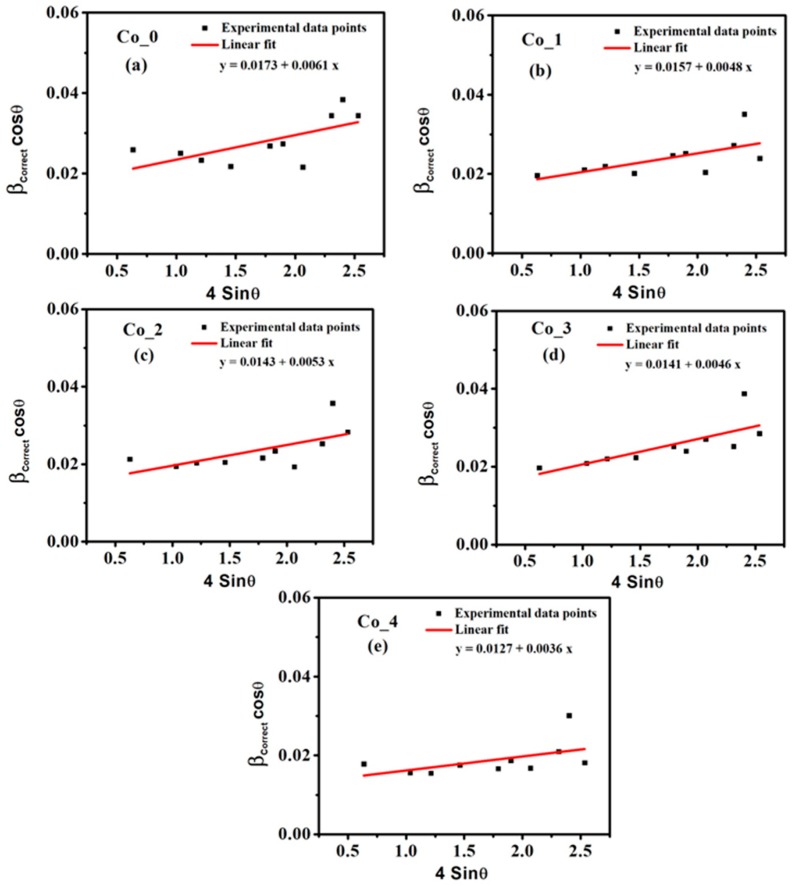
The Williamson–Hall (W–H) plots for the present CoxZn1-xFe2O4 ferrite systems (**a**) x = 0.00, (**b**) x = 0.10, (**c**) x = 0.20 and (**d**) x = 0.30, (**e**) x = 0.40.

Equation (4) is a linear regression between (*β_Correct_ cos θ*) and (*4 sin θ*), where the *D_L_* and ***ε_L_*** may be calculated from the intercept and the slope, as illustrated in Figure 2.

As reported in Table 1, the increase of the Co doping ratio from 0% to 40% increases the size of crystallites from 8.37 nm to 11.41 nm and decreases the micro-strain values from 6.1 × 10^−3^ to 3.6 × 10^−3^. Thus, an inverse relationship between the crystallite size (*D_L_*) and micro-strain (***ε_L_***) exists. The quality of the sample crystals is estimated by calculating the dislocation density (*δ_L_*). The *δ_L_* values of the samples was determined using the relation [35,36]

(5) $$\delta_{L}=\frac{1}{D_{L}{}^{2}}$$

Table 1 states that the dislocation density decreased with increasing the Co doping content. This behavior may be related to the improvement of the crystallization process because of Co-doping. The decrease of micro-strain leads to the increase in the crystallites size and the decrease of the dislocation density, as shown in Table 1. Co doping promotes the agglomeration of the small crystallites, that is, the coalescence of adjacent particles. Thus, the vacancies at the grain boundaries were shrunk. This in turn reduces the crystal defects of the synthesized Co_ (0, 1, 2, 3 and 4) samples including the micro-strain and dislocation density [37].

The lattice parameter (*a*) of Co_x_Zn_1-x_Fe_2_O_4_ spinel ferrite samples was calculated for the prominent peak (311) using the following equation [38]:

(6) $$a=d_{hkl}\sqrt{h^{2}+k^{2}+l^{2}}$$

We observed that the lattice parameter depends on the Co-doping ratio, which monotonically decreases with increasing of the Co-doping content as reported in Table 1. This Co doping dependence of the lattice parameter is ascribed to the smaller ionic radius of the cobalt atom (0.58 Å) compared to the zinc atom (0.6 Å) [36]. Ultimately, the addition of more Co improves the crystallinity and reduces the crystal defects of the synthesized Co_ (0, 1, 2, 3 and 4) samples.

### 3.2. Electron Microscopy Analysis

**Figure 3:**
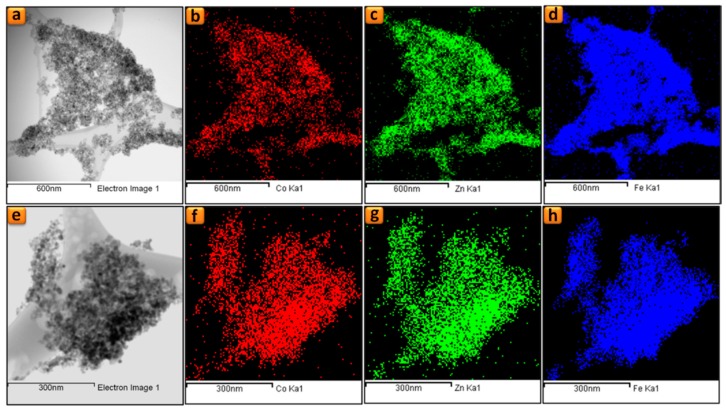
Elemental mapping for Co_2 (**a**–**d**) and Co_4 (**e**–**h**) samples: (**a**,**b**) BF-STEM images, (**b**,**f**) Co maps, (**c**,**g**) Zn maps and (**d**,**h**) Fe maps.

In this work, we utilized SEM and EDXS techniques to analyze the chemical composition, the morphology and the particles size distribution of Co_(0, 1, 2, 3 and 4). The EDXS spectra and elemental mapping (Figure S2 and Figure 3) emphasize the homogeneous dispersion of Co in Co_(0, 1, 2, 3 and 4) samples.

**Figure 4:**
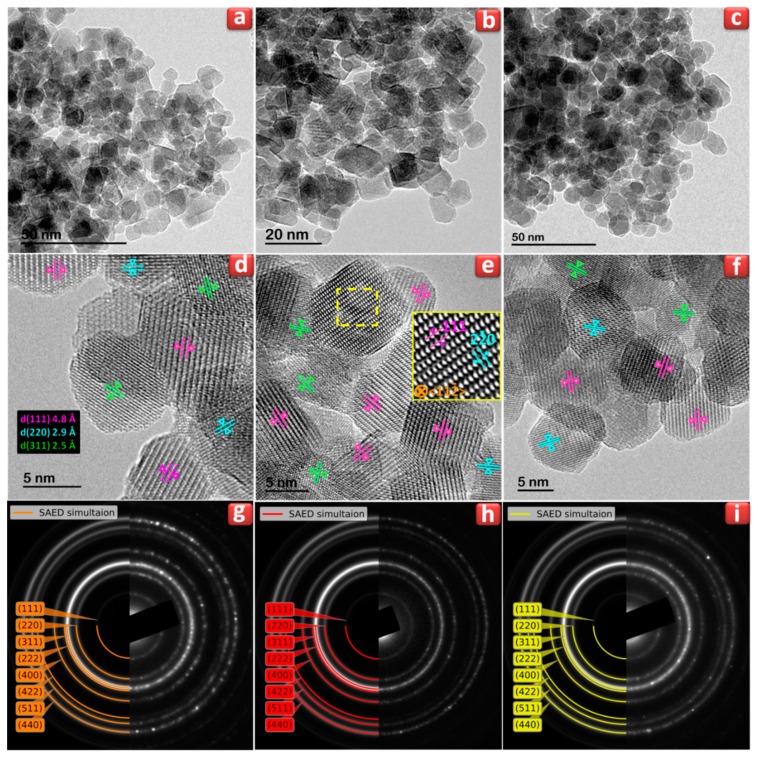
(**a–c**) TEM images; (**d–f**) HRTEM images; and (**g**–**i**) selected area electron diffraction (SAED) patterns (right) with the simulated ones (left) for Co_0, Co_2 and Co_4 samples.

SEM analysis verified the pure Co_(0, 1, 2, 3 and 4) sample nanoparticles had a sphere-like morphology at sizes below 20 nm, which increase as Co doping content increases (Figure S3). The Co_x_Zn_1-x_Fe_2_O_4_ nanoparticles distribution is mainly uniform and an agglomeration, due to particles magnetic dipole–dipole interactions, is observed [39]. The undoped ZnFe_2_O_4_ (Co0) and cobalt doped Co_x_Zn_1-x_Fe_2_O_4_ samples with x = 0.2 (Co_2) and 0.4 (Co_4) were selected for TEM studies. TEM micrographs (Figure 4a–c) of Co_0, Co_2 and Co_4 samples show the ~10 nm diameters of spherical particles.

Figure S4 displays histograms of sample particle-sizes for Co_0 (a), Co_2 (b) and Co_4 (c) estimated by binning greater than 200 particles. The solid blue line corresponds to the Log-normal distribution function fit with a mean size of 8.4(2), 9.8(1) and 10.2(1) nm and a variance of 0.30(2), 0.26(1) and 0.25(1), respectively. Apparently, the increase of Co^2+^ content leads to an increase of the particle’s size. The HRTEM micrographs shown in Figure 4d–f for the Co_0, Co_2 and Co_4 samples, indicate the superior crystallinity of these nanoparticles. In addition, analysis of Figure 4d–f concludes an interplanar spacing of 4.8 Å, 2.9 Å and 2.5 Å corresponding to (111), (220) and (311) atomic planes of ZnFe_2_O_4_ phase, respectively. The patterns of the selected area electron diffraction (SAED) in Figure 4g–i confirms the polycrystallinity of the samples under study. The pattern of all the rings have been indexed with reflections corresponding to space group *Fd-3m*. Moreover, all the SAED patterns are in excellent agreement with the simulated patterns from Zn-ferrite crystal structure data.

**Figure 5:**
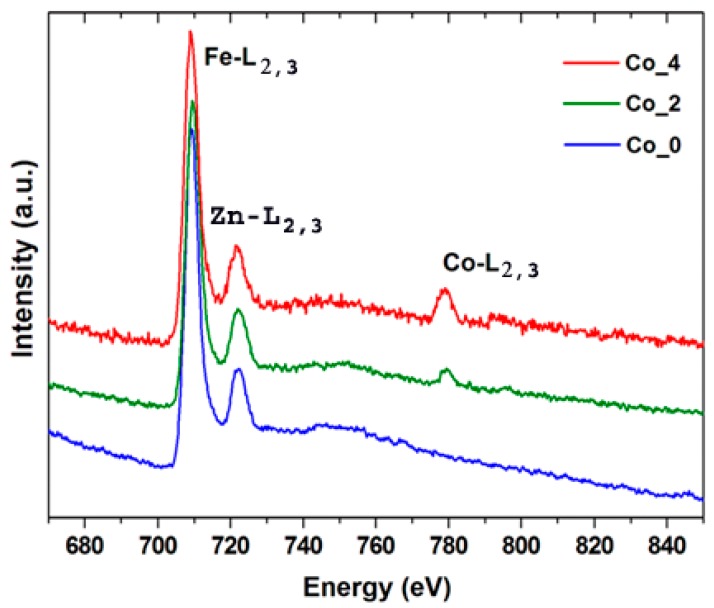
EELS spectra for **Co_0**, **Co_2** and **Co_4** samples.

The elemental and compositional properties of the Co_2 and Co_4 samples have been investigated by energy-dispersive X-ray spectroscopy (STEM-EDS) and electron energy loss spectroscopy (EELS). A successful and uniform doping of CO^2+^ in the synthesized compounds is verified by the STEM-EDS qualitative analysis (Figure S5 and Figure 5). The EELS spectra (Figure 5) unfolds strong Fe-L_2,3_ (2p_1/2_,2p_3/2_) edges for the Co_0, Co_2 and Co_4 samples. The EELS spectra of Figure 5 emphasize the monotonic increase in the edge intensity as the Co content increases.

Quantitative analysis of the STEM-EDS and EELS (Table 2) summarizes the expected general formula for the Co_2 and Co_4 samples as Co_0.2_Zn_0.8_Fe_2_O_4_ and Co_0.4_Zn_0.6_Fe_2_O_4_, respectively.

### 3.3. Magnetic Properties

**Figure 6:**
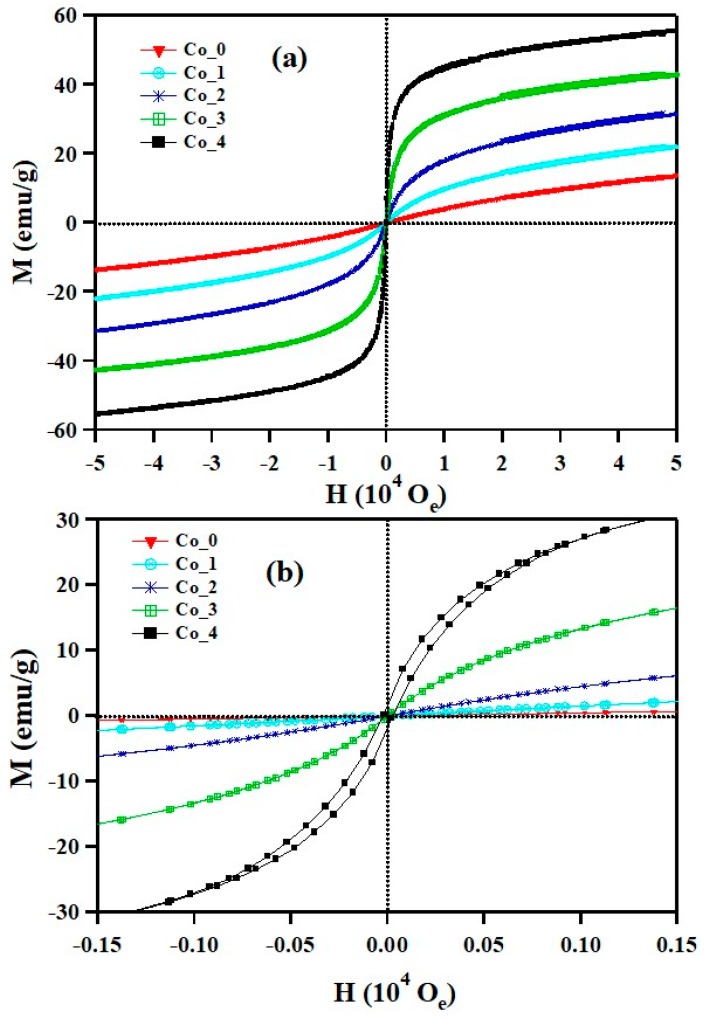
(**a**) Room temperature (300 K) magnetization-field (M-H) hysteresis loops of Co_x_Zn_1-x_Fe_2_O_4_ ferrite systems; (**b**) the magnification of the central area of the hysteresis loops.

The magnetic behavior of the Co_x_Zn_1-x_Fe_2_O_4_ ferrite systems (x = 0, 0.1, 0.2, 0.3 and 0.4) were measured using a vibrating sample magnetometer (VSM) within the range of ± 50,000 Oe. Figure 6 illustrates the change in the hysteresis curve on the Co^2+^ doping ratio at 300 K. The increase in the maximum magnetization, that is, M_m_, occurred from 14 emu/g to 55 emu/g with an increase of Co^2+^ content from 0% to 40%. For x = 0.4, the maximum magnetization, M_m_, equals the saturation magnetization, M_s_. The hysteresis behavior of ZnFe_2_O_4_, the parent compound of our investigated systems, are impacted by the addition of Co^2+^ dopant. For the Co_0 and Co_1 samples, the magnetization curves have a semi-linear representation which indicates a paramagnetic state. For Co_2 and Co_3, the samples have a sigmoidal response but show no hysteresis, which suggests the presence of a saturated superparamagnetic component [40,41,42,43]. Sample Co_4 displayed a weak ferromagnetic behavior, as shown in Figure 6b with a small remanence (1.46 emu/g) and coercive fields (27 Oe). Figure 6 also shows the response of the superparamagnetic compounds to the external field as a sigmoidal behavior as the ferromagnetic materials with minimal to no remanence and coercive components [44]. The preceding results indicate an important phase transition from paramagnetic to ferromagnetic state by increasing the cobalt content in the ZnFe_2_O_4_ host lattice.

The magnetic behavior of Co_(0, 1, 2, 3 and 4) samples at 300 K correlates with the crystallite size. The particle size of the Co_x_Zn_1-x_Fe_2_O_4_ ferrite systems increases from 8.3 nm to 11.4 nm with the increase of the Co^2+^ doping content as verified by structural analysis. When the particle size is small enough, the coercive field will become equal to zero and the remanent magnetization will disappear. Consequently, these materials respond as a paramagnetic compound [43,44]. The concept of super-paramagnetism is very close to that of ferromagnetism, the difference is the coherent strength of the magnetic domains. To have superparamagnetic materials, the crystal size must be sufficiently smaller than a Weiss domain of the corresponding bulk material. This ensures that the material form a single crystal domain in which they acquire uniform high magnetization with all the spins aligned in the same direction. In short, the bigger particles tend to show ferromagnetic state and smaller particles tend to show paramagnetic or superparamagnetic state.

**Figure 7:**
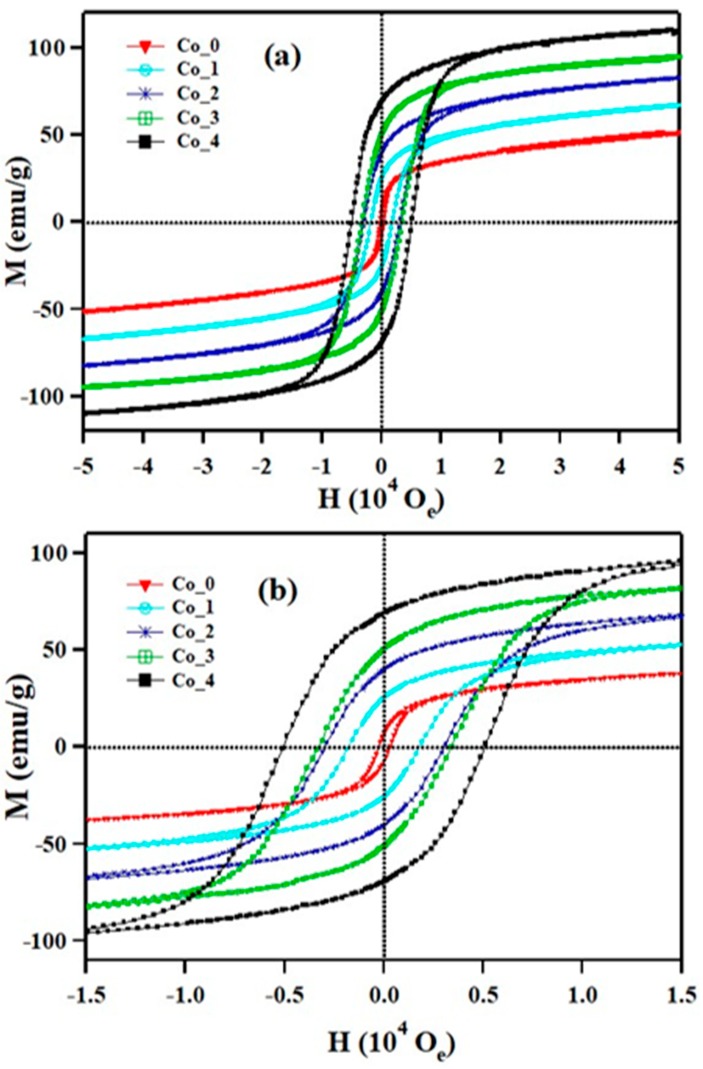
Magnetic field dependence of the magnetization measured at (**a**) 5K (M–H loops) of Co_x_Zn_1-x_Fe_2_O_4_ ferrite systems; (**b**) the magnification of the central area of the hysteresis loops.

Figure 7 illustrates the magnetization vs. applied magnetic field (M–H) in the range of ± 50,000 Oe at 5 K for Co_(0, 1, 2, 3 and 4) samples. The magnetization curves of all samples at 5K have a sigmoidal shape with a visible hysteresis loop (Figure 7b) which is characterized by ferromagnetic material [25].

The magnetic parameters, such as coercivity (H_C_), remanent magnetization (M_r_), squareness (M_r_/M_s_), saturation magnetization (M_s_) and anisotropy constant (k) were calculated from the hysteresis loops and listed in Table 3. The large values of coercive field and remanent magnetization are associated with the influence of the cationic stoichiometry and their positioning in specific lattice sites [22]. At 5 K, the remanent magnetization for the samples ranges from 7.3 emu/g to 69 emu/g. Moreover, the coercivities ranges from 0.3 KOe to 5.1 KOe, as shown in Table 3. The ranges of the remanent magnetization and the coercivities indicate that the Co_x_Zn_1-x_Fe_2_O_4_ ferrite systems are paramagnetic-superparamagnetic at room temperature but is magnetically ordered at very low temperatures, for example, a ferromagnetic-like moment.

The saturation magnetization, M_s_, is found to increase with increasing Co^2+^ content from 40 emu/g for x = 0.0 to 98 emu/g for x = 0.4. In a cubic structure of spinel ferrites, the saturation magnetization originates from the differential magnetic moments of the metal ions at the octahedral (B) and tetrahedral (A) sites [24,25,26]. In ZnFe_2_O_4_, the nonmagnetic Zn^2+^ cations (0 μB) preferably occupy the (A) sites and the Fe^3+^ ions (5 μB) prefer the (B) sites. CoFe_2_O_4_ has an inverse spinel structure in which the highly magnetic Co^2+^ bivalent cation occupies B site and Fe^3+^ ions are distributed equally between A and B sites with their spins in the opposite direction. At the tetrahedral (A) and octahedral (B) sites, the magnetic moments of Fe^3+^ are mutually neutralized. Thus, the resultant moment of the ferrite compound is practically equivalent to the aggregate magnetic moments of Co^2+^ ions (3 μB) at the B sites. Neel’s model suggests that the saturation magnetization is determined by the super-exchange interactions of the two sublattices [45]. Substituting the nonmagnetic Zn^2+^ cations by magnetic Co^2+^ ions leads to the introduction of Co^2+^ ions into A sites; thus, increasing the super-exchange interactions of the A and B sites, which in turn leads to an increased magnetization. The anisotropy value (*k*) for all samples reads [46]

(7) $$k=\frac{M_{S}H_{C}}{0.96}.$$

The variation of anisotropy value with increasing Co^2+^ content is reported in Table 3. We observed that the increase of Co^2+^ content in the ZnFe_2_O_4_ host lattice has a significant impact on the magnetic anisotropy (*k*). The increase in the anisotropy constant with Co^2+^ content reveals that the magnetic dipoles have a stronger alignment in each direction [15,39].

**Figure 8:**
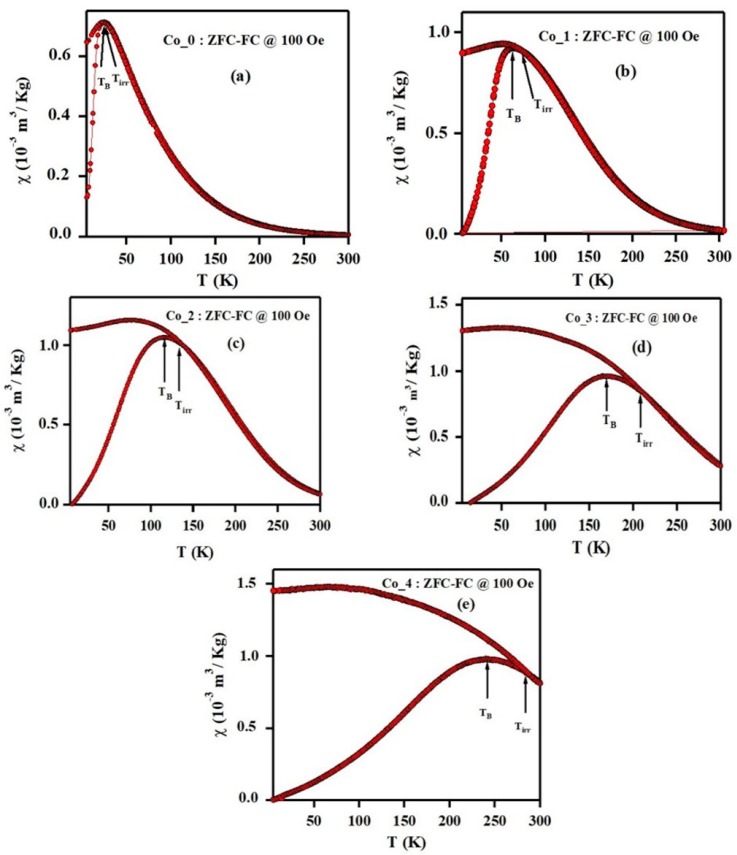
ZFC-FC magnetization curves measured with H = 100 Oe for Co_x_Zn_1-x_Fe_2_O_4_ ferrite systems (**a**) x = 0.00, (**b**) x = 0.10, (**c**) x = 0.20 and (**d**) x = 0.30, (**e**) x = 0.40.

The squareness ratio (M_r_/M_s_) could be used as a functional parameter for evaluating the homogeneity on dimension of the nanoparticles and the limit of single-domain-size of the magnetic nanomaterials [38]. The nature of magnetic domain in Co_x_Zn_1-x_Fe_2_O_4_ ferrite systems having a particular size can be calculated from the squareness (M_r_/M_s_) value. The single-domain nanosized ferromagnetic material could potentially be superparamagnetic at M_r_/M_s_ = 0. When M_r_/M_s_ does not equal zero, the prepared nanosized compound has both single-domain and multiple domain sizes. The larger particle size gives a higher M_r_/M_s_ ratio and in this case the ferromagnetic behavior becomes predominant. The values of M_r_/M_s_ for all Co_x_Zn_1-x_Fe_2_O_4_ ferrite samples were found to be in the range of 0.18 to 0.7, which suggests the formation of ferromagnetic states due to large particles in size distribution. Zero field-cooled (ZFC) and field-cooled (FC) magnetization curves for Co_(0, 1, 2, 3 and 4) samples were measured with an external field of 100 Oe over a wide temperature range (5K ≤ T ≤ 300 K), as illustrated in Figure 8.

The ZFC magnetization curves are typically obtained in two stages. First, in the absence of external magnetic field, we cool down the sample from 300 K (most particles show paramagnetic or superparamagnetic behavior) down to 5 K. In the second stage we apply a magnetic field of 100 Oe, while we increase the temperature from 5 K to 300 K in a stepwise manner. On the other hand, the FC magnetization curves are obtained by measuring the magnetization (M) while decreasing the temperature in the presence of a magnetic field [47]. The physical property we are interested in is the blocking temperature (T_B_), that is, the temperature at which maximum magnetization is achieved. The blocking temperature is sensitive to the grain-size distribution and indicates the transition between the superparamagnetic state (T > T_B_) and the blocked state (T < T_B_) [48]. Another feature of interest is the splitting or irreversibility temperature, T_irr_, which is qualitatively defined as that temperature where the ZFC and FC curves visibly diverge.

For a perfect superparamagnetic system (SPM) with a monodisperse particle size distribution, the irreversibility temperature will occur near the blocking temperature, that is, T_irr_ ~T_B_. However, for a superparamagnetic system with a finite size distribution the irreversibility temperature occurs at temperature larger than the blocking temperatures, that is, T > T_B_. Testa et al. [49] attributed the presence of a maximal turning point on ZFC curve for magnetic spinal ferrites to the blocking of the magnetic moment of individual particles and the mutual magnetic interactions between particles. As shown in Figure 8, there is an increase in the blocking temperature with particle size. Notably, the ZFC peaks shift towards the higher temperatures with increasing of Co^2+^ dopant ratio, indicating a continuous increase in the average grain size, which agrees with the structural analysis by XRD and TEM [50]. In the same magnetic field, larger particles experience blocking at higher temperature, whereas smaller particles does not seem to behave likewise. The larger volume of particles causes an increase in the anisotropy energy, which reduces the tunneling through the anisotropy barrier and hence the blocking inclines to higher temperatures [51].

As seen in Figure 8a,b, the ZFC magnetization curve of Co_0 and Co_1 samples (x = 0.0 and 0.1) reach a maximum value at the blocking temperature T_B_ equals to 25 K and 62 K, respectively. Beyond the blocking temperature, the magnetization decreases and shows a typical paramagnetic behavior. In addition, above the blocking temperature the FC magnetization curves follow the same path as ZFC curves. Furthermore, the rapid fall off of the ZFC curve peak for the Co_0 and Co_1 samples and the closeness of T_B_ and T_irr_ values illustrates the narrow size distribution for corresponding samples [49,50,51,52].

For the Co_2, Co_3 and Co_4 samples (x = 0.2, 0.3 and 0.4), the divergence of ZFC and FC curves at a certain irreversibility temperature is one of the characteristic features of superparamagnetic state. In other words, above the T_irr_ point, the Co_(2, 3 and 4) ferromagnetic clusters are fully unblocked. It is worth noting that the large difference between the blocking and the irreversibility temperatures indicates a homogeneous distribution of magnetic anisotropy in the sample. The maximum value of the ZFC curve at T_B_, shifts towards higher temperatures as the Co^2+^content increases, that is, T_B_ = 25 K, 62 K, 114 K, 170 K, and 240 K for Co_0, Co_1, Co_2, Co_3 and Co_4, respectively. Since the phenomenon of SPM blocking is related to magnetic anisotropy, the increasing of blocking temperature can be attributed to an increased particle anisotropy [53].

On comparing the FC–ZFC data and hysteresis curves data of our samples under study, we found that, below blocking temperature, the field-dependent magnetization of the Co_(0, 1, 2, 3 and 4) samples show the formation of pronounced hysteresis loops and hence behaves as ferromagnetic material. Above the blocking temperature, the hysteresis becomes infinitesimal, and the material behaves as paramagnetic or superparamagnetic material depending on the size of material particles.

## 4. Conclusions

Co_x_Zn_1-x_Fe_2_O_4_ spinal ferrite nanoparticles with different Co^2+^ ratios were successfully synthesized via the precipitation and hydrothermal method. This joint method of preparation has shown many advantages such as good physical homogeneity, a narrow distribution of particle size and high production rates. X-ray diffraction analysis validates that all the synthesized products were remarkably pure, and they exhibit single-phase cubic structures. The results show an increase in the crystallite size and decreases in both micro-strain and lattice parameter values, corresponds to an increased amount of Co^2+^ ions in the ZnFe_2_O_4_ host lattice. SEM and TEM microstructure analysis confirm the sphere-like morphology with particle sizes at 10 nm in diameters. HRTEM and SAED analysis validates the highly crystalline nature of all Co_x_Zn_1-x_Fe_2_O_4_ samples. Quantitative analysis of EDXS and EELS measurements, shows that the theoretical and measured elemental stoichiometry are in good agreement with each other. The magnetization measurements, at room temperature for the samples (x = 0 and 0.1), reveals the existence of unsaturated magnetization. The infinitesimal width of hysteresis loops for the samples with lower cobalt content (x = 0 and 0.1) implies a paramagnetic behavior. Increasing the cobalt doping (x = 0.2 and 0.3) transforms the magnetic phase from paramagnetic to superparamagnetic. The Co_x_Zn_1-x_Fe_2_O_4_ nanoparticles with x = 0.4 indicate saturated magnetization and finite hysteresis loop, which exhibits a weak ferromagnetic phase at room temperature. Ferromagnetic ordering with clear hysteresis behavior is experienced for all samples at low temperature (5K). The structural and magnetic properties of Co_x_Zn_1-x_Fe_2_O_4_ spinel ferrite nanoparticles are greatly influenced by the Co^2+^ doping ratio and temperature. The findings of current study help in optimizing the magneto-structural characteristics of ZnFe_2_O_4_ nanoparticles for many applications, such as spintronics and magnetic hyperthermia.

## Figures and Tables

**Table 1:** The calculated values of the crystallite size (*D_L_*), the micro-strain (*ε_L_*), the dislocation density (*δ_L_*) and the lattice parameter (*a*) of Co_ (0–4) ferrite systems.

Sample	*D_L_* (nm)	*ε_L_* × 10^−3^	*δ_L_* × 10^−3^ Line per cm^−2^	*a* (Å)
Co_0	8.378	6.1	14	8.454
Co_1	9.231	4.8	11.7	8.447
Co_2	10.135	5.3	9.7	8.446
Co_3	10.279	4.6	9.4	8.440
Co_4	11.412	3.6	7.6	8.431

**Table 2:** Quantitative analysis results in atomic % obtained from EDS and EELS measurements for Co_2 and Co_4 samples.

Sample	Element	Theoretical	STEM-EDS	EELS
**Co_2**	Fe Zn	66.7 26.7	65.6 27.1	66.5 -
	Co	6.6	7.3	6.8
**Co_4**	Fe Zn	66.7 20.0	66.2 18.5	65.8 -
	Co	13.3	15.3	14.2

**Table 3:** Coercivity (H_C_), remanent magnetization (M_r_), saturation magnetization (M_S_), squareness ratio (M_r_/M_S_), anisotropy constant (*k*), blocking temperature (T_B_) and irreversibility temperature (T_irr_) of Co_x_Zn_1-x_Fe_2_O_4_ ferrite systems.

Sample	H_C_ (Oe)	M_r_ (emu/g)	M_S_ (emu/g)	M_r_/M_S_	*k* (10^3^ erg/g)	T_B_ (K)	T_irr_ (K)
Co_0	300	7.3	40	0.18	12.5	25	24.6
Co_1	1900	25.5	55	0.46	108.8	62	73
Co_2	3100	40	71	0.56	229.3	114	133
Co_3	3400	51	84	0.61	297.5	170	209
Co_4	5100	69	98	0.70	520.6	240	284

## Supplementary Materials

The following are available online at https://www.mdpi.com/2079-4991/9/11/1602/s1, Figure S1: Diffraction peak profiles showing the experimental data (black solid line) and the fitting model (green lines for the individual peaks and red line for the cumulative model); a Lorentzian model was used to fit the individual diffraction peaks for all Co_(0–4) samples; Figure S2: SEM images and EDS spectra for Co_0 (a,b), Co_2 (c,d) samples and Co_4 (e,f); Figure S3: SEM images for the prepared doped ZnFe_2_O_4,_ Figure S4: Histograms of particle-size distribution for Co_0 (a), Co_2 (b) and Co_4 (c); Figure S5: Bright-field STEM images for Co_2 (a) and Co_4 (b) samples; yellow lines in (a,b) show line-scan profiles for Co (red), Zn (green) and Fe (blue) elements measured along a set of nanoparticles; Table S1: The position of observed XRD angles (*2θ)*, the calculated interplanar spacing (d_hkl_), the miller indices (hkl) according to JCPDS data and the calculated pure broadening (*β _Correct_*) according to Lorentzian distributions of Co_x_Zn_1-x_Fe_2_O_4_ ferrite systems.
