# Supplementary material for: Impact of Co2+ Substitution on Microstructure and Magnetic Properties of CoxZn1-xFe2O4 Nanoparticles
Source: Nanomaterials (Basel). 2019 Nov 11;9(11):1602. doi: 10.3390/nano9111602 (PMC6915397; doi:10.3390/nano9111602)
Supplement: Supplementary file 1 [file nanomaterials-09-01602-s001.pdf]

nanomaterials

Electronic Supporting Information

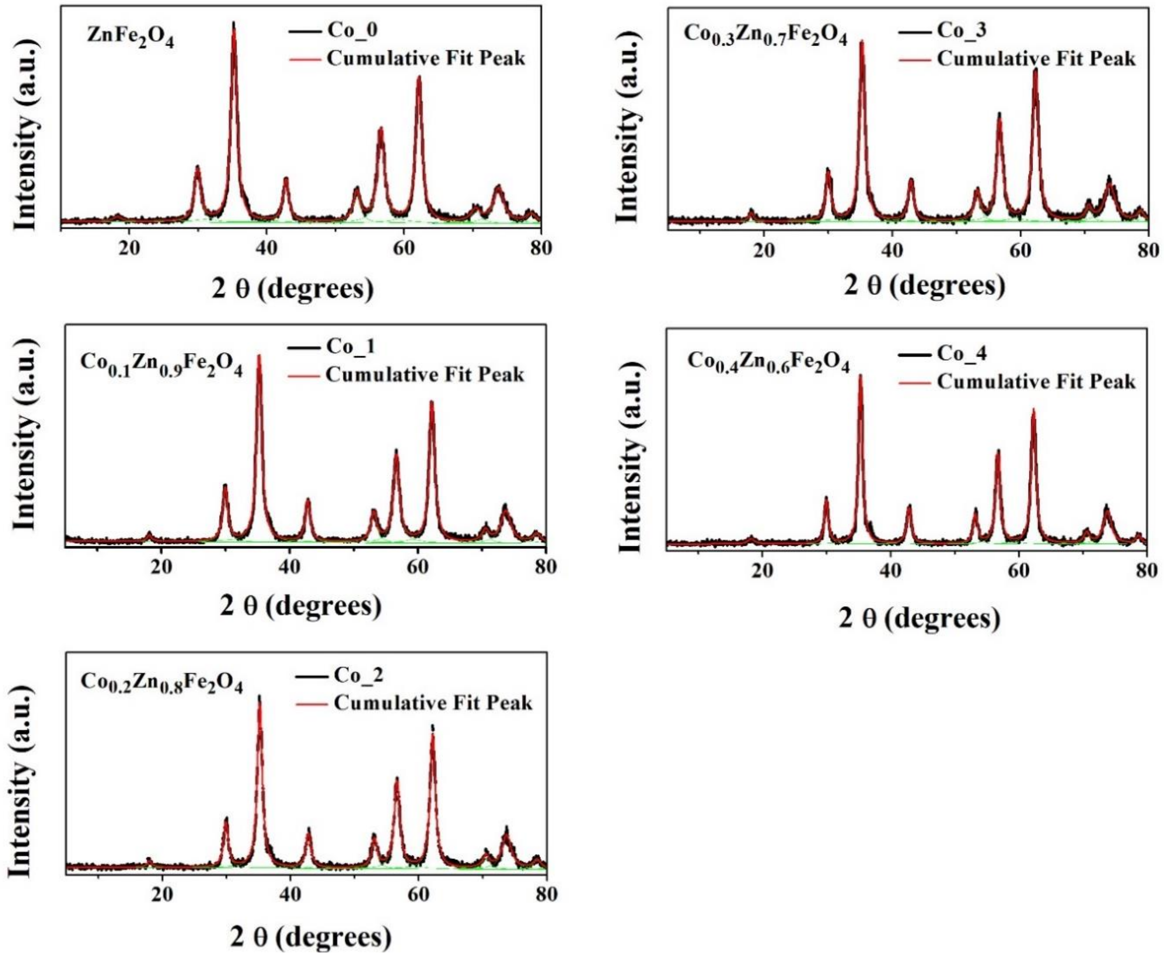

Figure S1. Diffraction peak profiles showing the experimental data (black solid line) and the fitting model (green lines for the individual peaks and red line for the cumulative model); A Lorentzian model was used to fit the individual diffraction peaks for all **Co_(0 – 4**) samples.

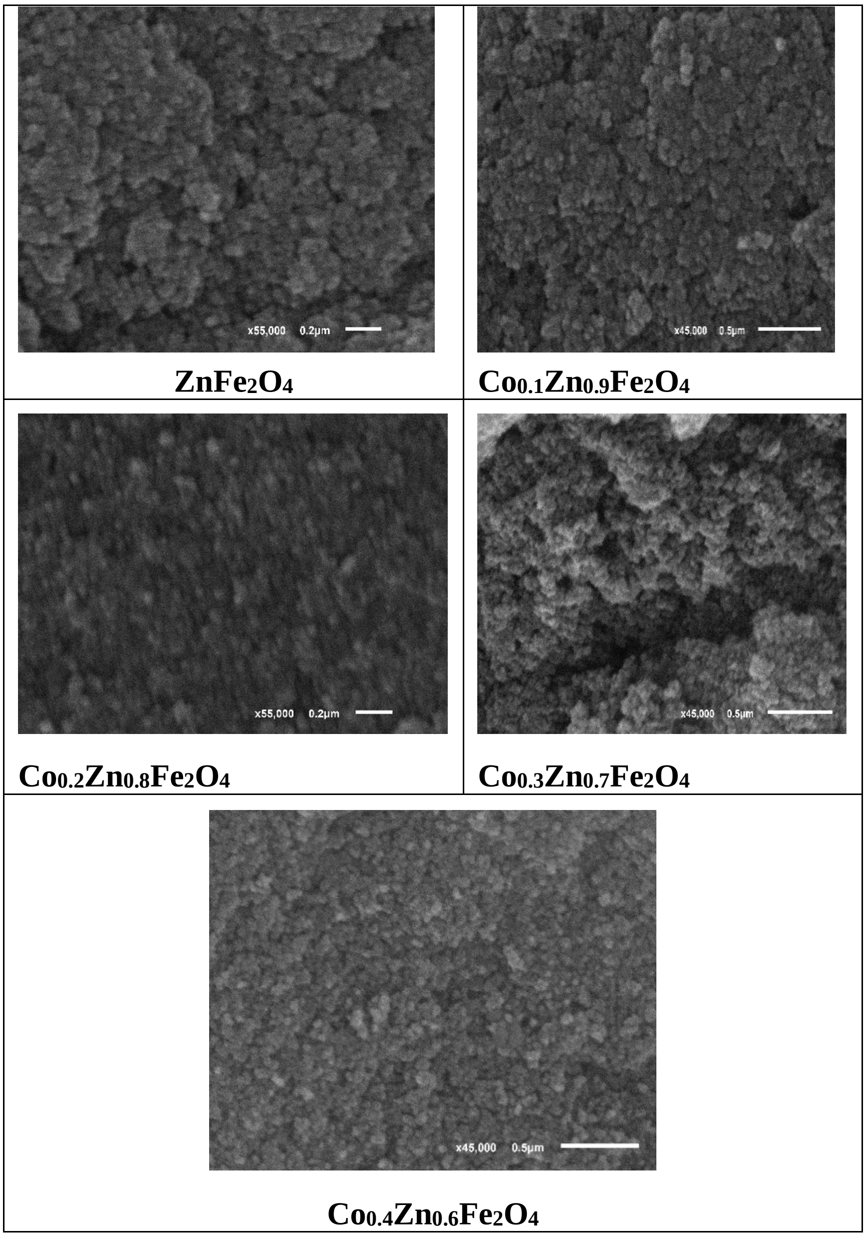

Figure S2. SEM images for the prepared doped $ZnFe_2O_4$.

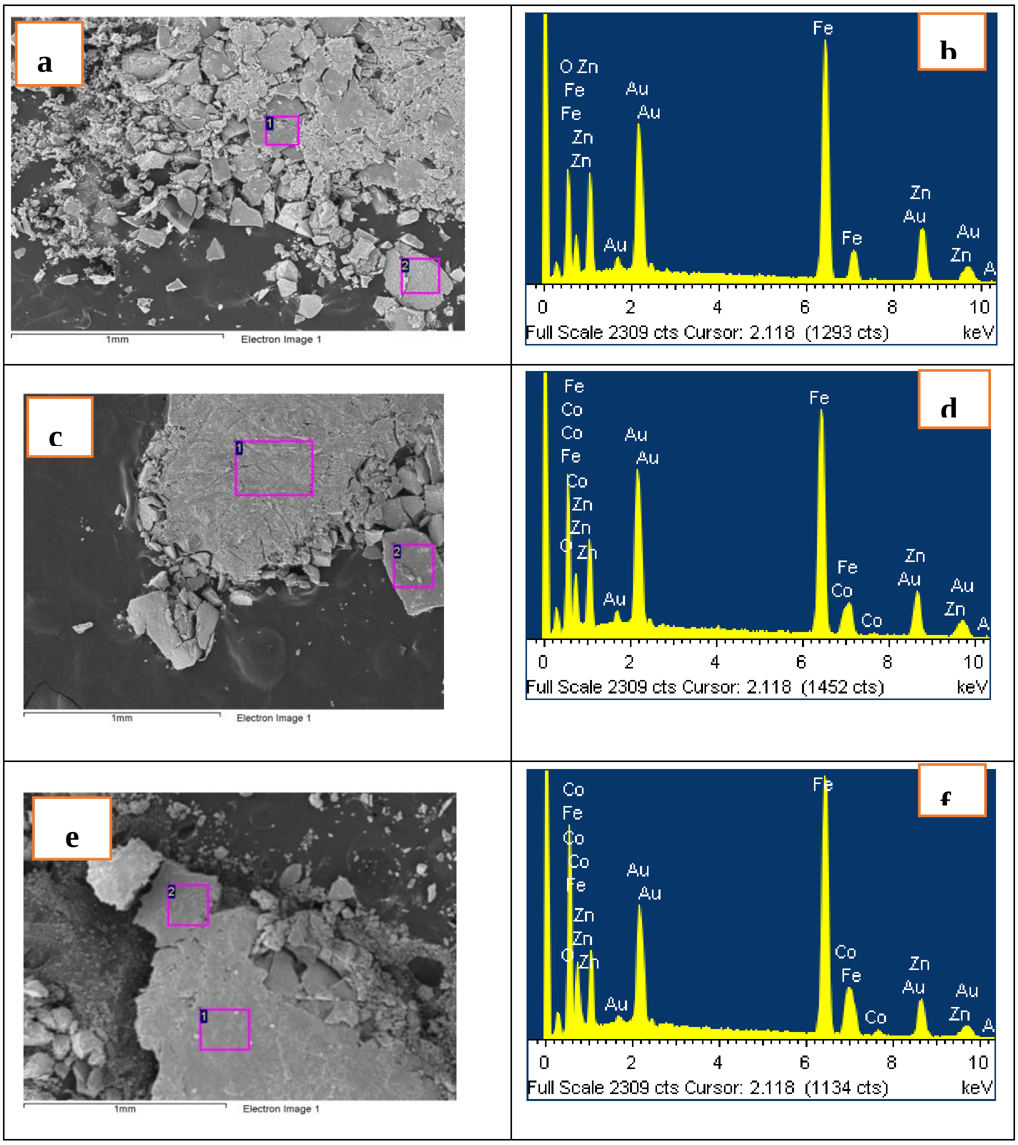

Figure S3. SEM images and EDS spectra for **Co_0** (a,b) , **Co_2** (c,d) samples and **Co_4** (e,f).

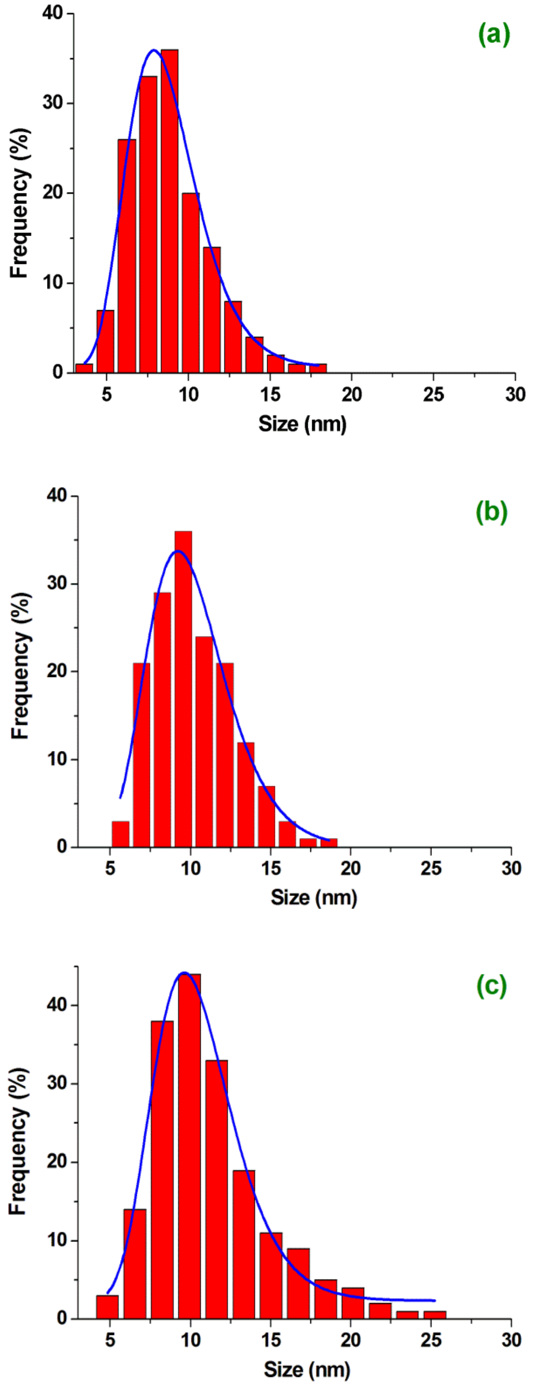

Figure S4. Histograms of particle-size distribution for **Co_0** (a), **Co_2** (b) and **Co_4** (c).

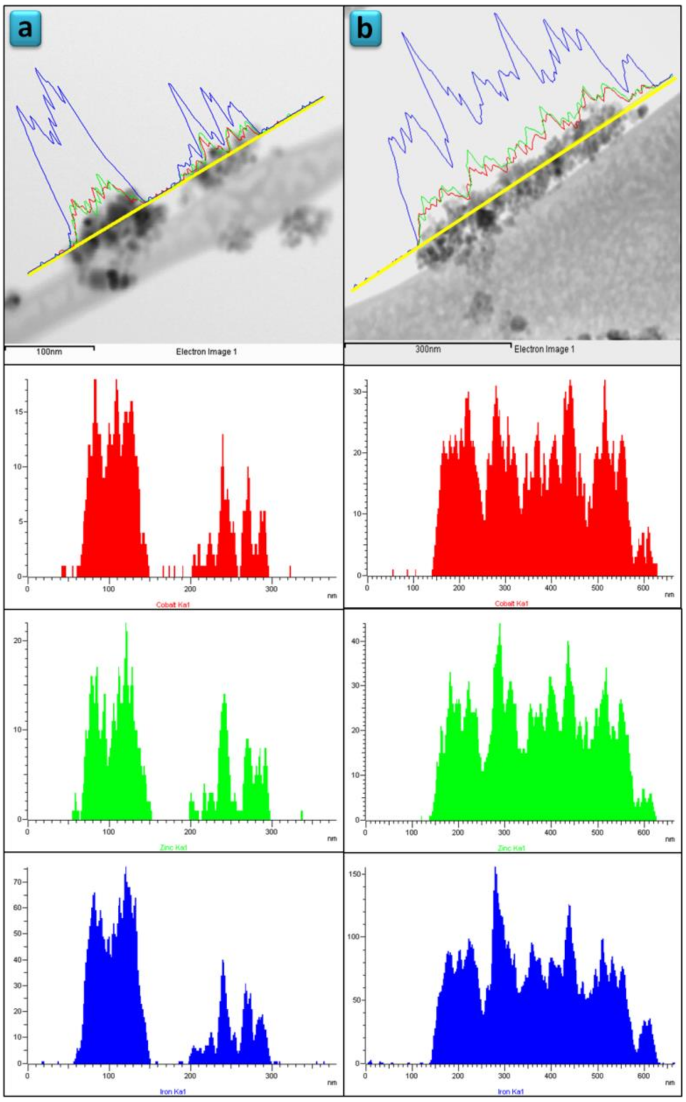

Figure S5. Bright-field STEM images for **Co_2** (a) and **Co_4** (b) samples; yellow lines in (a,b) show line-scan profiles for Co (red), Zn (green) and Fe (blue) elements measured along a set of nanoparticles.

Table S1. The position of observed XRD angles (**2*θ***), the calculated interplanar spacing (d_{hkl}), the miller indices (***hkl***) according to JCPDS data and the calculated pure broadening ($\beta_{Correct}$) according to Lorentzian distributions of $Co_xZn_{1-x}Fe_2O_4$ ferrite systems.

Samples	2 θ (deg.)	d_{hkl} (Å)	(hkl)	$\beta_{Correct}$ (deg.) Lorentz
$ZnFe_2O_4$	18.237	4.864	(111)	1.5035
	29.922	2.986	(220)	1.4838
	35.205	2.549	(311)	1.3999
	42.757	2.114	(400)	1.3364
	53.069	1.725	(422)	1.7201
	56.602	1.626	(511)	1.7817
	62.126	1.494	(440)	1.4407
	70.391	1.337	(620)	2.4140
	73.724	1.285	(533)	2.7456
	78.489	1.218	(444)	2.5435
$Co_{0.1}Zn_{0.9}Fe_2O_4$	18.093	4.902	(111)	1.1406
	29.922	2.986	(220)	1.2478
	35.232	2.547	(311)	1.3165
	42.799	2.112	(400)	1.2424
	53.102	1.724	(422)	1.5782
	56.618	1.625	(511)	1.6393
	62.169	1.493	(440)	1.3668
	70.502	1.335	(620)	1.9069
	73.705	1.285	(533)	2.5139
	78.503	1.218	(444)	1.7713
$Co_{0.2}Zn_{0.8}Fe_2O_4$	18.001	4.927	(111)	1.2361
	29.893	2.988	(220)	1.1546
	35.185	2.551	(311)	1.2225
	42.779	2.113	(400)	1.2626
	53.055	1.726	(422)	1.3864
	56.586	1.626	(511)	1.5239
	62.157	1.493	(440)	1.2944
	70.464	1.336	(620)	1.7774
	73.682	1.285	(533)	2.5594
	78.451	1.219	(444)	2.0968
$Co_{0.3}Zn_{0.7}Fe_2O_4$	17.936	4.945	(111)	
	29.977	2.981	(220)	1.1438
	35.261	2.545	(311)	1.2384
	42.863	2.109	(400)	1.3256
	53.202	1.721	(422)	1.3756
	56.702	1.623	(511)	1.6135
	62.298	1.490	(440)	1.5638
	70.592	1.334	(620)	1.8096
	73.873	1.282	(533)	1.7704
	78.691	1.2159	(444)	2.7784
$Co_{0.4}Zn_{0.6}Fe_2O_4$	18.284	4.852	(111)	1.0364
	29.975	2.981	(220)	0.9282
	35.299	2.542	(311)	0.9319
	42.865	2.109	(400)	1.0833
	53.204	1.721	(422)	1.0675
	56.719	1.622	(511)	1.2208
	62.279	1.491	(440)	1.1232
	70.588	1.334	(620)	1.4734
	73.804	1.283	(533)	2.1609
	78.661	1.2163	(444)	1.3451
